# Virtues and Proximate Elements of Ergot

**Published:** 1834

**Authors:** 


4. *Virtues and proximate elements of Ergot.*—Dr. Hooker, of New Haven, in the Boston Journal, announces that he has discovered in this article, an oil which possesses narcotic properties, and does not, in the slightest degree, promote the uterine contractions in parturition. Without attempting to cite his proofs, we may state that he disapproves of the use of Ergot in substance, and lays down the two following rules:

1. *The ergot should always be exhibited in the form of a watery infusion.*
2. *A large quantity should not be exhibited in any case—the infusion of twenty-five or thirty grains, in divided doses, being abundantly sufficient.*

In an obstetrical practice of some extent, we have seldom found it necessary to resort to this article, and are strongly inclined to believe, that in most cases in which it has been given, a patient waiting would have been preferable.

